# Inter-examiner reliability in periodontal diagnosis and treatment planning using the 2018 classification and the EFP S3 clinical guideline

**DOI:** 10.1186/s12903-026-09560-4

**Published:** 2026-08-17

**Authors:** Ahmet Dogukan Gundogdu, Vecihe Merve Balta-Uysal, Esra Guzeldemir-Akcakanat

**Affiliations:** 1 Faculty of Dentistry, Department of Periodontology, Kocaeli University, Kocaeli, Türkiye; 2 Private Practice, Dubai, UAE; 3 QU Health, College of Dental Medicine, Qatar University, Doha, Qatar

**Keywords:** Classification, Diagnosis, Periodontitis, Periodontal therapy, Reproducibility of results

## Abstract

**Background:**

The 2018 Classification of Periodontal and Peri-Implant Diseases and Conditions introduced a multidimensional diagnostic framework incorporating staging, grading, and extent, the European Federation of Periodontology (EFP) S3-level clinical practice guidelines provided structured treatment recommendations. Although these frameworks aim to improve diagnostic precision and therapeutic standardization, their inter-examiner reliability in routine clinical settings remains insufficiently validated. This study evaluated diagnostic and treatment planning agreement between clinicians with different levels of experience.

**Methods:**

In this single-center cross-sectional inter-examiner reliability study, 148 recruited patients diagnosed with periodontitis were included. Comprehensive clinical and radiographic records were obtained for each patient. Two blinded examiners, a first-year periodontology resident and an experienced periodontist, independently classified stage, grade and extent according to the 2018 Classification, and developed treatment plans based on the EFP S3 Clinical Practice Guidelines. Inter-examiner agreement was assessed using Cohen’s kappa (κ) with statistical significance set at *p* < 0.05.

**Results:**

Inter-examiner agreement for staging was moderate (κ = 0.463), whereas grading (κ = 0.899) and extent (κ = 0.816) demonstrated almost perfect agreement. Complexity-related variables showed variable reliability, with substantial agreement for flaring (κ = 0.715) but lower kappa values for bite collapse (κ = 0.231) and drifting (κ = 0.116), influenced by low prevalence. Treatment planning agreement was perfect for supragingival biofilm control and diabetes management (κ = 1.000) and almost perfect for smoking cessation counseling (κ = 0.973). Moderate agreement was observed for subgingival instrumentation (κ = 0.487), adjunctive chemotherapeutic agents (κ = 0.543), systemic antibiotics (κ = 0.519), resective surgery (κ = 0.549), and regenerative surgery (κ = 0.603).

**Conclusions:**

Risk-based components of the 2018 Classification demonstrate high reproducibility, whereas staging, particularly differentiation between Stage III and Stage IV, remains challenging. Treatment decisions for non-surgical therapy are more consistent than those involving surgical or adjunctive interventions. Enhanced calibration and structured decision-support tools may improve uniformity in advanced case management.

**Supplementary Information:**

The online version contains supplementary material available at https://doi.org/10.1186/s12903-026-09560-4.

## Introduction

Oral health is an integral and essential component of overall well-being and general health [1]. One of the key aspects of oral health is periodontal health [2]. According to the Global Burden of Disease (GBD) study, 3.69 billion people worldwide were affected by oral diseases [3]. Among oral diseases, periodontal diseases are non-communicable and preventable conditions [4]. Periodontal diseases are characterized as chronic inflammatory conditions that affect the tooth supporting tissues [5]. Risk factors for periodontal diseases, such as poor oral hygiene, smoking, and diabetes, are well established. Periodontitis is a prevalent chronic inflammatory disease affecting the supporting tissues of the teeth and is driven by dysbiotic dental biofilms in interaction with host-related risk factors such as smoking and diabetes mellitus. According to the recent GBD Study, severe periodontitis has increased substantially over recent decades, highlighting the importance of accurate diagnosis and timely treatment [3]. Accurate periodontal diagnosis is essential for disease prevention, appropriate referral, and evidence-based treatment planning, thereby improving long-term outcomes. Classification systems provide a common framework for disease diagnosis, professional communication, research, and evidence-based treatment planning [6]. Rather than replacing clinical judgment, they support consistent decision-making and facilitate standardized patient management across different clinical settings [6]. The 2018 Classification of Periodontal and Peri-Implant Diseases and Conditions introduced a new framework for the classification of periodontitis that replaced the previous distinction between chronic and aggressive periodontitis, recognizing them as a continuum influenced by severity, complexity, progression rate, and extent [7]. The framework characterizes periodontitis according to stage, grade and extent. Staging (I–IV) reflects disease severity and management complexity, grading (A–C) estimates the risk and rate of progression based on established modifying factors, and extent describes disease distribution [8]. Although this framework has improved diagnostic precision, individualized treatment planning, and professional communication, its multidimensional structure, which integrates clinical findings, radiographic assessment, tooth loss due to periodontitis, and systemic or behavioral risk modifiers, may introduce interpretative variability in daily practice, particularly in clinically complex cases or diagnostic “gray zones”.[9–12] Consequently, the consistent application of the 2018 Classification depends not only on clearly defined diagnostic criteria but also on adequate examiner calibration and clinical experience.

Previous investigations have reported variable levels of agreement in applying the 2018 Classification across different educational and professional backgrounds [9–13]. However, most studies were conducted using preselected or hypothetical case scenarios presented in survey format [9–11, 13]. Consequently, evidence derived from larger, consecutively recruited clinical cohorts that more closely reflect routine patient populations remains limited. Case-based survey designs, in which a fixed set of pre-documented vignettes is distributed to examiners, inherently exclude much of the diagnostic uncertainty encountered in daily practice, including patients with overlapping comorbidities, borderline or “gray-zone” presentations, and a naturally heterogeneous distribution of disease severity. Consequently, reliability estimates generated under such controlled, pre-selected conditions may not adequately capture the variability that arises when clinicians evaluate consecutively recruited, unselected patients in real time.

Periodontal treatment planning was based on the EFP S3-level Clinical Practice Guidelines, which recommend a structured stepwise approach including behavioral modification and risk factor control, non-surgical periodontal therapy, surgical intervention when indicated, and supportive periodontal care [14, 15]. Because therapeutic recommendations are directly linked to disease stage, complexity, and risk profile, accurate diagnosis is fundamental; misclassification may lead to inappropriate treatment intensity, resulting in either undertreatment of advanced disease or unnecessary overtreatment of less severe cases.

Limited studies have evaluated the 2018 Classification in well-established cohorts, particularly inter-examiner dynamics between trainees and specialists. This study addresses this gap by comparing a periodontology resident and experienced specialist in periodontics, assessing diagnostic and treatment consistency based on the 2018 Classification of Periodontal and Peri-Implant Diseases and Conditions [7], and the subsequently formulated treatment plans based on The EFP S3 level clinical practice guidelines [14, 15]. Given the importance of clinician experience in periodontal diagnosis, evaluating agreement between examiners with different levels of training represents a clinically meaningful approach to understanding variability in real-world practice.

Within this context, the main objective of the present study is to compare the consistency between periodontitis diagnoses and established treatment plans by two clinicians with different levels of experience in a large patient group, based on the 2018 Classification of Periodontal and Peri-Implant Diseases and Conditions and the EFP S3 clinical practice guideline.

## Methods

This single-center cross-sectional inter-examiner reliability study with prospective data was conducted at the Department of Periodontology Faculty of Dentistry Kocaeli University between 2022 and 2023. The study employed a fully prospective design, in which all clinical data, diagnoses, and treatment plans were generated in real time. Patients were consecutively recruited following their initial clinical triage appointment. The study recruitment flow is presented in Figure S1.

The trial design was approved by the Ethics Committee of the Medical Faculty of Kocaeli University which was conducted in compliance with the Declaration of Helsinki (GOKAEK-2022/16.08). Written informed consent was obtained from all participants.

### Intra-examiner calibration

A comprehensive clinical examination and documentation were performed by a periodontology resident examiner (ADG). Prior to commencing main data collection, intra-examiner reliability was assessed by comparing two independent assessments performed by the resident examiner on 20 randomly selected patients with periodontitis for clinical examination [16, 17]. These cases were not included in the main study. The time interval between assessments was two weeks to minimize recall bias.

Agreement for each parameter was quantified using Cohen’s kappa (κ) coefficient, and percent agreement was calculated as a supplementary measure. Kappa values were interpreted according to the criteria proposed by Landis and Koch (1977): κ < 0.20 = slight, 0.21–0.40 = fair, 0.41–0.60 = moderate, 0.61–0.80 = substantial, and κ > 0.80 = almost perfect agreement.

The resident examiner demonstrated almost perfect intra-examiner reliability for continuous clinical measurements demonstrated almost perfect reliability (ICC > 0.90).

In addition, both examiners independently underwent intra-examiner calibration exercises using 10 patients with periodontitis for all periodontal diagnostic parameters and treatment planning. These cases were not included in the main study and were selected from the clinic archive. Each examiner re-evaluated the same cases after a minimum interval of two weeks.

For the resident examiner, almost perfect agreement was observed for the majority of variables between repeated assessments, reflecting excellent intra-examiner reproducibility in periodontal diagnosis and treatment planning (κ = 1.00 for most parameters). Resective periodontal surgery demonstrated substantial agreement (κ = 0.783; percent agreement = 90.0%), while staging showed moderate agreement (κ = 0.524; percent agreement = 80.0%), with two discordant cases. For tooth drifting, percent agreement was high (90.0%); however, the kappa value was 0.000, reflecting the known prevalence paradox, where highly imbalanced category distributions inflate expected agreement and reduce kappa values.

For the experienced periodontist (VMBU), intra-examiner calibration for diagnostic and treatment planning parameters demonstrated almost perfect agreement for the majority of variables (κ = 1.00), with a mean kappa value of 0.934. Resective periodontal surgery showed substantial agreement (κ = 0.737; percent agreement = 90.0%), with one discordant case. Similarly, for bite collapse, high percent agreement (90.0%) was accompanied by a kappa value of 0.000, again attributable to the prevalence paradox. The experienced periodontist demonstrated almost perfect intra-examiner reproducibility across the large majority of parameters (mean κ = 0.934), supporting the reliability of the diagnostic and treatment planning data recorded in the study.

Overall, intra-examiner calibration demonstrated high reproducibility for both examiners, supporting the reliability of the recorded clinical data and subsequent diagnostic and treatment planning assessments.

To minimize potential bias, no diagnosis or treatment planning was performed at the time of clinical data collection. All clinical measurements, photographs, and records were obtained first, and diagnostic assessment and treatment planning were conducted independently by each examiner after completion of data collection for all patients. The study workflow is illustrated in Figure S1.

### Sample size calculation

Based on previous literature (Table 1), sample size calculation for inter-examiner reliability using Cohen’s kappa assumed a null hypothesis kappa value of 0.50 and an expected kappa of 0.70. With a type I error rate of α = 0.05 and 80% power, the minimum required sample size was 128 patients. A total of 148 patients were included to account for potential data loss.

**Table 1:** Overview of reliability studies on the 2018 periodontal classification and treatment guidelines

Study	Case	Examiner	Conduction of the study	Provided data	Stage	Grade	Extent	Treatment Planning	Supportive periodontal therapy
Abou-Arraj et al. 2021 [13]	10 cases	181 Participants - Second-year predoctoral dental students(*n* = 76) vs. Fourth-year predoctoral students(*n* = 76) vs. Orthodontics postgraduate students(*n* = 17) vs. Periodontology postgraduate student(*n* = 12) Gold standard two experienced periodontists	Survey	Medical and dental history Intraoral photographs Radiographs (OPG, bitewing and periapicals) Full mouth periodontal chart	For all diagnosis κ 0.24	-	-	Prophylaxis κ0.23 Scaling and root planing therapy κ0.63 Periodontal resective surgery κ0.33 Periodontal regenerative surgery κ0.20 Extraction of hopeless teeth κ0.36 Complex multidisciplinary treatment κ0.23	κ 0.44
Jayawardena et al. 2021 [18]	75 referral cases	Referee vs. Specialist periodontists	Referral letters	Pocket depth (six point) Radiographs	κ 0.197 50.7%	κ 0.158 57.3%	-	-	-
Marini et al. 2021 [11]	25 periodontitis cases	30 Participants - Undergraduate students (*n* = 10) vs. general dentist (*n* = 10) vs. specialist periodontist (*n* = 10) vs. gold-standard examiner	two slideshow presentation	Medical/dental history intra-oral photographs full-mouth periapical radiographs Full mouth periodontal chart	κ 0.48 76.0%	κ 0.45 71.4%	κ 0.37 82.6%	-	-
Ravida et al. 2021 [10]	9 severe periodontitis cases	Periodontist (*n* = 103) vs. 5 gold-standard experts	Online survey	Age, sex, medial history, intraoral photos, full mouth periodontal charts, smoking status, diabetes status, bitewing and periapical radiographs.	κ 0.49 76.6%	κ 0.5 82%	κ 0.51 84.8%	-	-
Abrahamian et al. 2022 [9]	5 periodontitis cases	174 Participants - (University faculty (*n* = 77) vs. specialist clinicians (*n* = 36) vs. postgraduate students (*n* = 61) vs. gold standart 7 experts	Online survey	Medical and dental history Intraoral photos, full mouth periodontal charting, OPG and full mouth periapical radiographs.	0.71 82.30%	0.85 91.40%	0.52 83%	-	-
Kakar et al. 2022 [19]	10 cases	81 participants - Full-time and adjunct (*n* = 20) vs. Full-time and adjunct (*n* = 20) vs. Non-academic full-time periodontist from private practice (*n* = 21) vs. Non-academic full-time general dentist (*n* = 20)	Online survey	Medical and dental history intra-oral photographs full mouth series radiographs periodontal charting (PD, CAL, recession, furcation, mobility)	overall diagnosis ranged from κ 0.36–0.55			overall treatment ranged from κ 0.32–0.42	
Gandhi et al. 2022 [12]	10 cases	Undergraduate students from different years and different schools (*n* = 263) vs. Gold standard (3 periodontists)	Online survey	Medical and dental history intraoral photographs, full mouth periodontal charting, full mouth radiographic series	overall diagnosis ranged from κ 0.03–0.25 25.2%			overall treatment ranged from κ 0.06–0.53 66.7%	
Kutuk et al. 2025 [20]	10 periodontitis cases	15 participants - Senior periodontist (*n* = 5) vs. Postgraduate periodontology students (*n* = 5) vs. Undergraduate dental students(*n* = 5) vs. Gold standard (two periodontists S3 EFP)	Survey	Age, gender, medical and medical history, systemic diseases, medication, smoking, dental history full periodontal charting, radiographs (periapical and/or panoramic)	κ 0.366 58.7%	κ 0.222 53.3%	κ 0.287 78%	Tooth extraction κ 0.554 Mechanical supragingival plaque/calculus removal κ -0.007 Subgingival Instrumentation κ -0.002 Resective periodontal surgery κ 0.255 Regenerative periodontal surgery κ 0.161 furcation involvement κ 0.31 Adjunctive therapies κ 0.06	κ 0.106
Ali et al. 2025 [21]	4 periodontitis cases	312 participants - General Dental Practitioners (*n* = 82) vs. Postgraduate Residents other (*n* = 21) vs. Postgraduate Residents in periodontics (*n* = 22) vs. Specialists in Periodontics (*n* = 64) vs. Undergraduate Dental Students (*n* = 88) vs. Other Specialists (*n* = 29)	Online survey	Medical/dental history, intra-oral examination, periodontal examination panoramic and full periapical radiographs, clinical images	Mean diagnostic overall 26.27%			-	-
Herrero et al. 2025 [22]	12 periodontitis cases	32 participants - Dental students (*n* = 12) vs. Dentists with up to 3 years of experience, general dentists (*n* = 10) vs. Dentists with > 4 years of experience with (*n* = 10) vs. Gold standard expert periodontists	Virtual classroom survey	Age, gender, dental/medical history (HbA1c, smoking), intraoral photographs, complete periodontal chart, full series of radiographs	κ = 0.561 (Group 2 vs. Group 1), κ = 0.451 (Group 3 vs. Group 2), and κ = 0.376 (Group 3 vs. Group 1)	κ = 0.387 (Group 3 vs. Group 2), κ = 0.338 (Group 2 vs. Group 1), and κ = 0.313 (Group 3 vs. Group 1)	κ = 0.552 (Group 2 vs. Group 1), κ = 0.436 (Group 3 vs. Group 1), and κ = 0.415 (Group 3 vs. Group 2)	-	-
Oh et al. 2021 [23]	3 periodontitis cases	64 participants - Dental practitioners with a periodontal background (*n* = 31) vs. Other educational backgrounds (*n* = 33)	Survey	Medical/dental history, full-mouth periodontal chart, intraoral clinical photographs, intraoral complete radiographs	κ = 0.34	κ = 0.34	-	-	-

### Participants

Following an initial diagnosis of periodontitis during triage, eligibility was confirmed through a structured screening process. All participants were fully informed about the study procedures and provided written informed consent prior to data collection.

A total of 148 consecutively recruited patients with detectable bone loss following clinical and radiographic screening were included in this study. Eligibility criteria comprised age ≥ 18 years, the presence of at least 20 natural teeth, including teeth exhibiting varying degrees of mobility, and availability of a recent orthopantomogram (OPG) and, if available, periapical radiographs.

Patients were excluded if they had received periodontal therapy or systemic antibiotic treatment within the previous six months, were pregnant or lactating, undergoing active orthodontic treatment, or had systemic conditions requiring antibiotic prophylaxis. Individuals with infectious or inflammatory systemic diseases, a history of hepatitis or HIV infection, immunosuppressive chemotherapy, and oral conditions or diseases other than periodontitis were also excluded.

### Clinical periodontal assessment

Before commencement of the study, both examiners completed intra-examiner calibration exercises using separate calibration cases that were not included in the final analysis. The purpose of this calibration was to ensure consistent application of the 2018 Classification and EFP S3 treatment recommendations before independent evaluation of the study cohort.

For each patient, a standardized case file was created, including sociodemographic variables (age, gender, and smoking status), detailed medical and dental history, full-mouth periodontal charting, orthopantomogram (OPG), and intraoral photographs.

A comprehensive full-mouth periodontal examination was conducted. Plaque index (PI), probing depth (PD), bleeding on probing (BOP), gingival recession (GR), and clinical attachment loss (CAL) were measured at six sites per tooth. Furcation involvement and tooth mobility were also assessed and documented.

A periodontology resident and an experienced specialist in periodontics were deliberately selected as the two examiners to model a clinically representative trainee–supervisor diagnostic relationship, a scenario commonly encountered in postgraduate periodontal training and specialist referral practice.

Upon completion of data collection, both examiners, independently, classified each case according to the 2018 Classification of Periodontal Diseases and Conditions [7], assigning stage, grade, and extent. Subsequently, individualized treatment plans were developed in accordance with the EFP S3-level clinical practice guidelines [14, 15]. Treatment planning followed the structured stepwise approach of the guideline: Step 1 (supragingival biofilm control by the patient and professionally delivered, and risk factor control including smoking cessation and glycemic management); Step 2 (subgingival instrumentation, with or without adjunctive chemical agents or systemically administered antibiotics); Step 3 (resective or regenerative periodontal surgery); and Step 4 (supportive periodontal care).

Each examiner performed all diagnostic and treatment planning assessments independently, and no consensus procedure was applied. This approach was intentionally adopted to avoid introducing bias and to preserve the validity of inter-examiner reliability measurements. Disagreements between examiners were not resolved, as the primary objective of the study was to quantify agreement rather than to establish a single correct diagnosis.

### Statistical analysis

Descriptive statistics (mean, standard deviation, minimum, maximum) were used to define continuous variables. The normality of continuous variables was assessed using the Shapiro-Wilk test. Inter-examiner agreement level was evaluated using the Kappa coefficient and percentage agreement was calculated as a complementary measure. For ordinal variables such as periodontal staging, weighted kappa statistics were also applied to account for the degree of disagreement between examiners.

The scale was interpreted as follows: ≤ 0 – no agreement; 0.01–0.20 - slight agreement; 0.21–0.40 – fair agreement; 0.41–0.60 moderate agreement; 0.61–0.80 substantial agreement, and 0.81–1.00 (almost perfect agreement) [24]. The level of statistical significance was set at 0.05.

All analyses were performed using MedCalc^®^ Statistical Software version 19.7.2 (MedCalc Software Ltd, Ostend, Belgium; 2021) and SPSS version 31 (IBM Corp., Armonk, NY, USA).

## Results

A total of 148 patients were included in the study (mean age 45.5 ± 9.0 years; range 24–70 years). The study population comprised 54.7% males (*n* = 81) and 45.3% females (*n* = 67). Among the participants, 44.6% were current smokers, whereas 55.4% were non-smokers. Of the smokers, 31.1% reported consuming more than 10 cigarettes per day (Table 2).

**Table 2:** Demographic, systemic, and clinical characteristics of the study population

Gender n (%)	Female	67 (45.3)
	Male	81 (54.7)
Age	mean ± SD	45.5 ± 9.0 years
	age range	24–70
Smoking status n (%)	Non-smoker	82 (55.4)
	< 10 cigarettes/day	20 (13.5)
	≥ 10 cigarettes/day	46 (31.1)
	No diabetes	131 (88.5)
Diabetes mellitus	Diabetes + HbA1c < 7	4 (2.7)
n (%)	Diabetes + HbA1c ≥ 7	6 (4.1)
	Diabetes + HbA1c unknown	7 (4.7)
Periodontal Examination (mean ± SD)	Plaque Index	1.9 ± 0.4
	Bleeding on probing (%)	36.4 ± 18.1
	Probing depth (mm)	3.4 ± 0.6
	Gingival recession (mm)	0.4 ± 0.3
	Clinical attachment loss (mm)	2.5 ± 1.4
Furcation Involvement (n tooth)		16
Tooth Mobility (n, %)	Class I	183 (2.5)
	Class II	126 (1.7)
	Class III	1 (0.0027)

The prevalence of type II diabetes mellitus was 11.5%. Among patients with diabetes, 4.7% were unaware of their HbA1c levels, 2.7% had HbA1c values below 7%, and 4.1% had HbA1c values ≥ 7% (Table 2).

Periodontal parameters assessed in this study are presented in Table 2. For periodontal staging, inter-examiner agreement was moderate (Cohen’s κ = 0.463; 95% CI: 0.291–0.635, *p* < 0.001), with an overall percentage agreement of 81.8%. Most classifications were concentrated in Stage III, with 118 and 115 cases identified by the specialist and resident, respectively. Disagreement was observed in 27 cases. Weighted kappa analysis using quadratic weighting demonstrated a comparable level of agreement (κw = 0.482), suggesting that discrepancies were primarily between adjacent stages rather than markedly different classifications, thereby supporting the robustness of the agreement despite minor variations in staging (Table 3).

**Table 3:** Summary of inter-examiner agreement for staging, grading, and bone loss/age

Variable	Agreement (%)	κ (95% CI)	Agreement Level
Stage	81.8	0.463 (0.291–0.635)	Moderate
Grade	95.3	0.899 (0.821–0.977)	Almost perfect
Bone loss/age	87.8	0.756 (0.650–0.862)	Substantial

A summary of agreement metrics for key variables is presented in Table 3, whereas detailed category-level results are provided in Supplementary Table (Table S1).

For disease grading, no patient was classified as Grade A by either examiner. Agreement was 89.7% for Grade B and 98.9% for Grade C, with an overall percentage agreement of 95.3%. Inter-examiner agreement was almost perfect (Cohen’s κ = 0.899; 95% CI: 0.821–0.977, *p* < 0.001). Weighted kappa analysis further supported this finding, demonstrating excellent consistency between examiners even when accounting for the magnitude of disagreement (Table 3).

When case phenotype was evaluated based on the relationship between biofilm accumulation and the level of periodontal destruction, inter-examiner agreement was substantial (κ = 0.785, *p* < 0.001). The specialist periodontist classified 2 patients as having heavy biofilm deposits with low levels of destruction, 121 patients as showing destruction commensurate with biofilm levels, and 25 patients as exhibiting destruction exceeding expectations relative to biofilm accumulation. Corresponding classifications by the resident were 5, 119, and 24 patients, respectively. Disagreement occurred in 10 cases (Table S1).

For percentage bone loss relative to age, inter-examiner agreement was substantial (Cohen’s κ = 0.756; 95% CI: 0.650–0.862), with a high percentage agreement of 87.8%. No patient was categorized below 0.25 by either examiner. The specialist periodontist classified 78 patients within the 0.25–1 range and 70 patients above 1, whereas the resident classified 80 and 68 patients in these categories, respectively. Disagreement was observed in 18 cases. Weighted kappa analysis demonstrated a similar level of agreement, suggesting that discrepancies were predominantly between neighboring categories rather than representing major classification differences (Table 3).

Regarding disease extent, examiners agreed in 138 cases, with disagreement observed in 10 patients. Statistical analysis demonstrated almost perfect agreement (κ = 0.816, *p* < 0.001) (Table 3).

Although observed agreement for bite collapse was high (89.9%, 133/148 patients), the kappa value was 0.231 (*p* = 0.005), indicating fair agreement. This discrepancy between high raw agreement (90%) and lower kappa likely reflects the highly imbalanced distribution of responses, as the vast majority of cases were classified as absence of occlusal collapse [25]. 

Similarly, for tooth drifting, overall observed agreement was 84.5% (125/148 cases), whereas the kappa coefficient was 0.116 (*p* = 0.005), corresponding to slight agreement. The low kappa value appears to be influenced by the very low prevalence of positive cases (2.0%), which increases the expected chance agreement and may artificially reduce the kappa statistic [25]. 

For tooth flaring, observed agreement was 95.3%, with concordant assessments in 141 of 148 cases. The kappa coefficient was 0.72 (*p* < 0.001), indicating substantial inter-examiner agreement.

Inter-examiner agreement for treatment planning decisions is summarized in Table 4. Complete agreement was achieved for Step 1 (patient-performed supragingival biofilm control, professionally delivered supragingival biofilm control, and diabetes management) and Step 4 periodontal therapy. Disagreement occurred in only two cases concerning smoking cessation counseling, resulting in overall high agreement for Step 1 interventions.

**Table 4:** Inter-examiner agreement for periodontal treatment planning

Treatment Steps		Agreement *n* (%)	Reliability (κ, *p*, and agreement level)
Step 1	Patient supragingival control	148 (100)	κ = 1.000, *p* < 0.001, Perfect
	Professional supragingival control	148 (100)	κ = 1.000, *p* < 0.001, Perfect
	Smoking	146 (98.65)	κ = 0.973, *p* < 0.001, Almost perfect
	Diabetes management	148 (100)	κ = 1.000, *p* < 0.001, Perfect
Step 2	Subgingival instrumentation	144 (97.30)	κ = 0.487, *p* < 0.001, Moderate
	Chemotherapeutics	126 (85.14)	κ = 0.543, *p* < 0.001, Moderate
	Systemic antibiotics	132 (89.19)	κ = 0.519, *p* < 0.001, Moderate
Step 3	Resective surgery	114 (77.03)	κ = 0.549, *p* < 0.001, Moderate
	Regenerative surgery	124 (83.78)	κ = 0.603, *p* < 0.001, Moderate
Step 4	Supportive periodontal therapy	148 (100)	κ = 1.000, *p* < 0.001, Perfect

In Step 2 therapy, disagreement was observed in 4 cases for subgingival instrumentation, 22 cases for adjunctive chemotherapeutic agent use, and 16 cases for systemic antibiotic prescription. Overall, agreement for Step 2 treatment options was moderate, reflecting greater variability in decisions involving adjunctive or systemic interventions. Percentage agreement was also calculated and demonstrated a similar pattern to the kappa statistics, with higher agreement for less complex treatment decisions and lower agreement for more complex treatment planning steps.

In cases of disagreement, subgingival instrumentation was preferred more often by the experienced examiner (*n* = 3) compared with the less experienced examiner (*n* = 1). Conversely, the less experienced examiner more frequently recommended systemic antibiotics (*n* = 11) and adjunctive chemotherapeutic agents (*n* = 16), whereas the experienced examiner recommended systemic antibiotics (*n* = 5) and chemotherapeutic agents (*n* = 6) less often.

In Step 3 treatment planning, resective surgery was recommended in 114 cases (77.03%), yielding a moderate level of inter-examiner agreement (κ = 0.549, *p* < 0.001). Regenerative surgery was proposed in 124 cases (83.78%), also demonstrating moderate agreement between examiners (κ = 0.603, *p* < 0.001). Disagreement between examiners was observed in 34 cases for resective surgery and in 24 cases for regenerative surgery. In cases of disagreement, the less experienced examiner more frequently recommended resective (*n* = 32) and regenerative (*n* = 16) surgical approaches, whereas the experienced examiner recommended resective (*n* = 2) and regenerative (*n* = 8) surgery less often.

## Discussion

The present study evaluated the inter-examiner reliability of the 2018 Classification of Periodontal and Peri-implant Diseases and Conditions and the consistency of treatment planning according to EFP S3 clinical guidelines between clinicians with differing levels of experience. The findings demonstrate moderate agreement for staging and high agreement for grading and extent. Treatment planning decisions were highly consistent for non-surgical periodontal treatment (plaque control and subgingival instrumentation) but more variable for surgical and adjunctive therapies.

Several studies have previously evaluated intra- and inter-examiner reliability in diagnosing periodontitis according to the 2018 Classification [9–13, 18, 20, 21, 26]. Most of these studies have adopted a case-based survey design, in which a limited number of standardized and fully documented periodontal disease and/or periodontitis cases were electronically distributed to the examiners from various clinical settings and with different experience levels including undergraduate students. Although this methodology allows controlled comparison across participants, it may not fully capture the complexity and variability encountered in routine clinical practice. By contrast, the present study reflects real-world diagnostic and treatment-planning conditions, incorporating consecutively recruited patients with the full clinical complexity of comorbidities, radiographic findings, and borderline presentations, conditions that pre-selected case vignettes cannot replicate.

Consistent with previous literature, staging emerged as the most challenging diagnostic component [9]. Earlier reliability studies have reported slight to fair [12, 13, 18–21, 23] and moderate [9, 10, 22] inter-examiner agreement for overall periodontitis diagnosis, including stage, grade and extent, with greater variability observed in advanced stages (III/IV) [9]. The moderate kappa value observed in the present study largely reflects difficulty in distinguishing between Stage III and Stage IV periodontitis. This distinction depends on complexity-related parameters, such as tooth loss attributable to periodontitis, masticatory dysfunction, and bite collapse, that require clinical judgment and may be interpreted differently depending on experience [9]. The existence of diagnostic `gray zones` within the staging framework has been acknowledged by prior investigations and remains a source of variability [10, 11]. 

In this study, notably, neither examiner diagnosed Stage I periodontitis. This likely reflects referral-related selection bias, as patients were triaged by general practitioners prior to inclusion and therefore tended to present with more advanced disease. While this limits the generalizability of the findings across the full spectrum of periodontal conditions, it also enabled focused evaluation of inter-examiner variability in moderate to advanced cases, where diagnostic and treatment planning discrepancies are more likely to occur. Marini et al. [11] reported that clinicians were better discriminating Stage III and IV than Stage I and II. This finding highlights the clinical challenge of differentiating early attachment loss from gingivitis, particularly when radiographic changes are subtle [27]. While this distinction may not substantially alter initial non-surgical periodontal therapy and risk factor control in routine practice, it is methodologically significant in research contexts, where accurate disease stratification influences epidemiological estimates and reliability outcomes.

Less experienced clinicians, such as undergraduate students and recent graduates, may either overestimate disease severity in early stages [13] or encounter difficulties when interpreting complexity-related parameters, including vertical bone loss, advanced furcation involvement, masticatory dysfunction and the assessment of teeth with questionable prognosis. However, Herrero et al. reported the opposite trend, observing underestimation of disease severity by the less experienced clinicians and overestimation by more experienced clinicians [22]. However, previous studies consistently indicated that periodontology specialists and postgraduate students demonstrate higher levels of agreement and accuracy in applying the 2018 Classification compared to general dentists and undergraduate students [13, 18, 19, 21]. Misdiagnosis or underestimation of disease severity at the primary care level may delay appropriate referral to specialist periodontists, potentially leading to disease progression and avoidable tooth loss. From a public health perspective, this represents a significant concern. Accordingly, encouraging the implementation of structured clinical guidelines, decision-support tools, and simplified flowcharts tailored for general dentists and dental hygienists may enhance early recognition and facilitate timely referral pathways [28, 29]. 

In contrast to staging, grading demonstrated almost perfect agreement. This finding suggests that clearly defined modifying risk factors, such as smoking intensity and glycemic control, enhance diagnostic reproducibility. When grading relies on objective and well-documented parameters, variability appears reduced. This observation aligns with studies reporting higher agreement for grading when risk indicators are explicitly available [9]. Conversely, when grading depends primarily on indirect measures such as bone loss–to–age ratios, interpretative differences may increase [20]. Marini et al. [11]and Kütük et al. [21] reported lower agreement in grading compared to staging, highlighting that reliability may depend on the clarity and availability of grading criteria within clinical records.

Regarding treatment planning, disagreements in diagnosis were not reflected to the same extent in therapeutic decisions, consistent with the literature [13]. Near-perfect agreement was observed for Step 1 interventions and supportive periodontal care, [14] suggesting that foundational non-surgical management follows a structured and largely algorithm-driven approach. However, moderate agreement for surgical procedures and adjunctive systemic or chemotherapeutic interventions indicates that advanced therapeutic decisions remain influenced by clinical experience and individual thresholds for intervention [13]. 

Notably, the less experienced examiner demonstrated a tendency toward more intervention-oriented decisions, including increased recommendations for systemic antibiotics and surgical procedures. This pattern may reflect a lower threshold for intervention, potentially driven by uncertainty in clinical decision-making or a more cautious approach to disease management. In contrast, the experienced periodontist appeared to adopt a more conservative and selective treatment strategy, likely based on greater familiarity with disease progression, long-term outcomes, and current evidence discouraging unnecessary adjunctive or surgical interventions.

These findings have important clinical implications, as they highlight how differences in experience can influence not only diagnostic agreement but also treatment strategies, potentially affecting patient outcomes. Over-prescription of antibiotics and increased inclination toward surgical interventions may have consequences in terms of antimicrobial resistance, patient burden, and healthcare costs. This variability appears to be driven not only by diagnostic uncertainty but also by differences in clinical decision thresholds between examiners, as previously reported in the literature, highlighting the influence of clinical experience on decision-making, particularly in cases requiring interpretation of borderline findings or “gray zones” within the classification system. This underscores the need for structured decision-support tools and clearer operational definitions for complexity-related parameters. Furthermore, even relatively small differences in staging may translate into different treatment pathways because therapeutic recommendations within the EFP S3 guideline are directly linked to disease severity and complexity.

The moderate agreement regarding adjunctive chemotherapeutic and systemic antibiotic use is particularly noteworthy. Current evidence discourages routine antibiotic prescription and supports selective use in specific clinical scenarios, such as Stage III, Grade C cases or young patients with rapid progression [30, 31]. Variability observed in this domain may reflect differences in risk perception and therapeutic philosophy rather than misunderstanding of the guideline itself. This underscores the importance of reinforcing evidence-based decision-making principles during postgraduate training.

A further methodological observation concerns complexity-related variables such as bite collapse and drifting. Despite high raw agreement, kappa values were reduced due to low prevalence, illustrating the known “prevalence paradox” associated with kappa statistics. This statistical nuance should be considered when interpreting reliability outcomes in conditions with skewed distributions.

Several limitations should be acknowledged. The study involved only two examiners, which may limit generalizability of the findings. However, this was a deliberate methodological choice (as described in Methods) aimed at reflecting a clinically relevant scenario commonly encountered in periodontal practice and postgraduate training, namely, the interaction between a trainee and a supervising specialist. Including a larger number of examiners would have increased between-examiner heterogeneity and made it more difficult to specifically evaluate the influence of clinical experience, which represented the primary objective of the present study. This dyadic design has been utilized in previous inter-examiner reliability studies and allows focused evaluation of experience-related variability. Furthermore, the relatively large number of cases included in this study (*n* = 148) provides a robust basis for assessing agreement across a wide spectrum of periodontal conditions, partially mitigating the limitation related to the number of examiners.

In addition, unlike previous studies that included multiple examiners but were based on a limited number of cases, the present study incorporated a substantially larger case set (*n* = 148), enabling a more comprehensive assessment across a broad spectrum of clinical presentations. Additionally, inclusion criteria requiring at least 20 natural teeth may have underrepresented extreme Stage IV cases. This threshold was established to ensure accurate case definition and minimize potential misclassification related to tooth loss of uncertain etiology. In clinical practice, determining whether missing teeth are exclusively attributable to periodontitis can be challenging, particularly in the absence of comprehensive historical documentation. Furthermore, it should be clarified whether teeth with a hopeless prognosis or those indicated for extraction due to severe mobility should be considered as already lost within the staging framework [32]. At the inclusion stage of the study, the presence of at least 20 teeth was taken into account; however, these specific situations were left to the independent diagnostic judgment of the examiners during the evaluation process.

The study population was skewed toward more advanced periodontal cases, with no Stage I cases included, likely reflecting the clinical referral pattern in which patients more commonly present with moderate to severe disease. While this may introduce selection bias and limit generalizability across the full disease spectrum, it enabled a focused evaluation of inter-examiner variability in more complex cases, where diagnostic and treatment planning discrepancies are more likely to arise, as early-stage cases typically involve more straightforward clinical decisions. Accordingly, the present findings should primarily be interpreted as applicable to patients with moderate to advanced periodontitis. Future multicenter studies involving primary care settings and a broader spectrum of disease severity, including Stage I periodontitis, are warranted to determine whether similar levels of diagnostic and treatment planning reliability can be achieved across the full spectrum of periodontal disease.

The absence of a criterion standard diagnosis is an inherent limitation of inter-examiner reliability studies, as agreement between examiners does not necessarily reflect diagnostic accuracy. Importantly, reliability studies are designed to assess consistency rather than validity. Introducing a consensus-based or third-examiner “gold standard” could artificially inflate agreement and shift the study design toward validity assessment rather than reliability. Future studies incorporating expert panel consensus or reference standards would be valuable to complement reliability findings with accuracy evaluation.

### Clinical implications

The present findings suggest that the 2018 Classification can be applied with high reproducibility for grading and disease extent in routine periodontal practice. However, differentiation between Stage III and Stage IV remains a clinically challenging area that may benefit from structured calibration, decision-support tools, and targeted postgraduate education. The greater variability observed in surgical and adjunctive treatment decisions highlights the importance of clinician experience and supports continued implementation of evidence-based training programs to improve consistency in patient management.

## Conclusion

The findings of the present study demonstrate high reproducibility for periodontal grading and disease extent, whereas differentiation between Stage III and Stage IV periodontitis remains the most challenging aspect of the 2018 Classification.

With respect to grading, the presence of clearly defined modifying risk factors, such as smoking and diabetes, appeared to reduce diagnostic ambiguity and improve inter-examiner agreement. In contrast, variability increased in their absence, suggesting that grading may be more susceptible to subjective interpretation when indirect evidence of progression is required.

Regarding treatment planning, high levels of agreement were observed for non-surgical periodontal therapy. Conversely, decisions related to surgical interventions and the use of systemic antibiotics or adjunctive chemotherapeutic agents, areas where recommendations require careful case-by-case evaluation, were more strongly influenced by clinical experience.

Overall, the results support the reproducibility of grading and extent assessment while identifying staging and advanced treatment decisions as areas requiring enhanced calibration.

## Supplementary Information

Supplementary Material 1. Table S1. Detailed inter-examiner agreement results for periodontitis staging, grading, extent, complexity- and prognosis-related variables.

Supplementary Material 2. Figure S1. Study flow chart

## Data Availability

The datasets generated during the current study are not publicly available but are available from the corresponding author on reasonable request.

## Abbreviations

GBD: Global Burden of Disease
CAL: Clinical Attachment Loss
EFP: European Federation of Periodontology
PI: Plaque Index
PD: Probing Depth
BOP: Bleeding on Probing
GR: Gingival Recession
OPG: Orthopantomogram
HbA1c: Glycated Hemoglobin (Hemoglobin A1c)
ICC: Intraclass Correlation Coefficient
HIV: Human Immunodeficiency Virus
κ / κw: Kappa / Weighted Kappa (Cohen's Kappa coefficient)
CI: Confidence Interval
ADG: Initials of the resident examiner
VMBU: Initials of the experienced periodontist examiner
