# Supplementary material for: Inter-examiner reliability in periodontal diagnosis and treatment planning using the 2018 classification and the EFP S3 clinical guideline
Source: BMC Oral Health. 2026 Aug 17;26:2241. doi: 10.1186/s12903-026-09560-4 (PMC13757175; doi:10.1186/s12903-026-09560-4)
Supplement: Supplementary file 1 [file 12903_2026_9560_MOESM1_ESM.docx]

**Table S1.** Detailed inter-examiner agreement results for periodontitis staging, grading, extent, complexity- and prognosis-related variables.

| **Parameters** | | **Agreement**  **(n, %)** | **Kappa (κ)**  **Agreement Level** | |  |
| --- | --- | --- | --- | --- | --- |
| **Stage** | **Stage I** | - | κ = 0.463, p < 0.001, Moderate | |  |
|  | **Stage II** | 1 (50) |  |  |  |
|  | **Stage III** | 103 (87.3) |  |  |  |
|  | **Stage IV** | 17 (60.7) |  |  |  |
| **Extent** | **Localized** | 21 (91.3) |  |  | |
|  | **Generalized** | 111 (94.9) | κ = 0.816, p < 0.001  Almost perfect | |  |
|  | **Molar-incisor** | 6 (75) |  |  | |
| **Bite collapse** | **yes** | 3 (27.3) | κ = 0.231, p = 0.005  Fair | |  |
|  | **no** | 130 (94.9%) |  |  |  |
| **Drifting** | **yes** | 2(8.3 %) | κ = 0.116, p = 0.005  Slight | |  |
|  | **no** | 123 (99.2) |  |  |  |
| **Flaring** | **yes** | 10 (66.7%) | κ = 0.715, p < 0.001  Substantial | |  |
|  | **no** | 131 (98.5) |  |  |  |
| **Grade** | **Grade A** | - | κ = 0.899, p < 0.001  Almost perfect | |  |
|  | **Grade B** | 52 (89.7) |  |  |  |
|  | **Grade C** | 89 (98.9) |  |  |  |
| **% Bone Loss/Age** | **0 – 0.25** | - | κ = 0.756, p < 0.001  Moderate | |  |
|  | **0.25 to 1** | 70 (89.7) |  |  |  |
|  | **>1.0** | 60 (85.7) |  |  |  |
| **Case Phenotype** | **Heavy biofilm deposits with low levels of destruction** | 2 (100) | κ = 0.785, p < 0.001  Moderate | |  |
|  | **Destruction commensurate with biofilm deposits** | 115 (95) |  |  |  |
|  | **Destruction exceeds expectation given biofilm deposits** | 21 (84) |  |  |  |
