# Supplementary material for: Inter-examiner reliability in periodontal diagnosis and treatment planning using the 2018 classification and the EFP S3 clinical guideline
Source: BMC Oral Health. 2026 Aug 17;26:2241. doi: 10.1186/s12903-026-09560-4 (PMC13757175; doi:10.1186/s12903-026-09560-4)
Supplement: Supplementary file 2 [file 12903_2026_9560_MOESM2_ESM.docx]

**Figure 1.** Study flow chart.

**INDEPENDANT**

**INDEPENDANT**

**Periodontology Resident**

Periodontal Diagnose

Periodontal Treatment Plan

**Experienced Periodontist**

Periodontal Diagnose

Periodontal Treatment Plan

**Experienced Periodontist**

Evaluated the periodontal chart, OPGs and intraoral photos

**Periodontology Resident**

Evaluated the periodontal chart, OPGs and intraoral photos

Patient Recruitment Completed (n=148)

A comprehensive full-mouth periodontal examination along with intraoral photographs was conducted and documented by Periodontology Resident.

**No periodontal diagnosis was established, no periodontal treatment plan**

**was formulated at this stage.**

✓

≥18 years age

At least 20 natural teeth

Patient Recruitment

Periodontology Resident (ADG)

DEPT OF PERIODONTOLOGY

Patients with a preliminary diagnosis of periodontitis referred after triage by general dentists

**DATA ANALYSES**
